# Familial investigation of two cases of pseudohypoparathyroidism

**DOI:** 10.3389/fendo.2026.1800965

**Published:** 2026-05-19

**Authors:** Huihui Yin, Yuenan Liu, Meixuan Li, Jiawen Li, Yang Yu, Jianling Du, Haicheng Zhou, Lili Men

**Affiliations:** Department of Endocrinology, The First Affiliated Hospital of Dalian Medical University, Dalian, China

**Keywords:** Albright’s hereditary osteodystrophy, *GNAS* gene mutation, hypocalcemia, inactivating PTH/PTHrP signaling disorders, pseudohypoparathyroidism

## Abstract

**Object:**

To report the clinical and genetic characteristics of patients diagnosed with pseudohypoparathyroidism (PHP) or inactivating parathyroid hormone (PTH)/PTHrP signaling disorders (iPPSD), and to provide insights for the diagnosis and management of this rare inherited metabolic disorder.

**Methods:**

This study focused on two patients diagnosed with PHP or iPPSD, aiming to investigate their clinical and genetic characteristics. Clinical manifestations and biochemical indicators of the two subjects were collected and analyzed. Whole-exome sequencing was employed to detect potential genetic mutations associated with the disorder. Additionally, pedigree analysis was performed to clarify the inheritance pattern of the identified mutation in the families of the two patients.

**Results:**

Both patients presented with recurrent seizures and Albright’s hereditary osteodystrophy (AHO) features. Biochemical tests revealed consistent abnormalities in both subjects, including hypocalcemia, hyperphosphatemia, and elevated levels of PTH and thyroid-stimulating hormone (TSH). Whole-exome sequencing successfully isolated a specific four-nucleotide deletion (GACT) within exon 7 of the *GNAS* gene, confirming the condition as iPPSD2. Pedigree analysis verified that the mutation was maternally inherited in both families; however, the clinical presentation of the mutation varied among relatives in each family.

**Conclusion:**

The symptoms of iPPSD are inconsistent and easily misidentified, which highlights the necessity of early genetic screening and PTH monitoring for patients with AHO-like physical traits or electrolyte imbalances. Precise molecular diagnosis is vital for ensuring effective and timely clinical intervention, as well as for preventing long-term complications associated with this rare metabolic disorder. This study provides valuable clinical and genetic data to enhance the understanding and management of PHP/iPPSD.

## Introduction

Pseudohypoparathyroidism (PHP) represents a complex group of rare metabolic disorders defined by a resistance to parathyroid hormone (PTH) at the level of target organs, specifically the bone and renal tubules (1, 2). This biochemical resistance typically manifests as a triad of hypocalcemia, hyperphosphatemia, and compensatory elevations in serum PTH (1, 3). Traditionally, PHP was classified into types I and II, with further subdivisions (Ia, Ib, and Ic) based on maternal versus paternal mutations in the GNAS gene, which encodes the alpha subunit of the stimulatory G protein (2, 4). These subtypes are caused by mutations in the maternal G protein alpha subunit (GNAS) gene whereas progressive osseous heteroplasia (POH) and pseudo- pseudohypoparathyroidism (PPHP) result from mutations in the paternal GNAS gene (5, 6). However, as our molecular understanding expanded to include related conditions such as progressive osseous POH and acrodysostosis (ACRDYS), the original nomenclature became increasingly inadequate (1). In 2018, a unified classification system was proposed, labelling these conditions as inactivating PTH/PTHrP signaling disorders (iPPSD) (1, 7). This modern framework categorizes the disorders by their specific genetic or epigenetic defects: iPPSD1 involves mutations in the PTHR1 receptor; iPPSD2 and iPPSD3 encompass GNAS protein defects or epigenetic silencing (formerly PHP-Ia and Ib); and iPPSD4 and iPPSD5 involve downstream signaling molecules like cAMP-dependent protein kinase or phosphodiesterase 4D (1).

The clinical presentation of iPPSD is highly variable and depends on the specific molecular subtype and the target organs affected (1, 8). Patients frequently suffer from symptoms of chronic hypocalcemia, including muscle cramps, circumoral numbness, and, in severe instances, laryngospasm or generalized seizures (1, 8). Over time, these metabolic imbalances can lead to dental hypoplasia, cataracts, and ectopic calcification within the basal ganglia (8, 9). Beyond mineral metabolism, patients, particularly those with iPPSD2, often exhibit the Albright’s hereditary osteodystrophy (AHO) phenotype, which includes short stature, brachydactyly (shortened fingers and toes), a rounded face, and subcutaneous ossifications (10). Many of these individuals also show varying degrees of resistance to other G-protein-coupled hormones, such as TSH, GHRH, and gonadotropins, leading to multi-endocrine deficiencies (1). Because the existing literature is heavily weighted toward isolated case reports, there is a significant clinical need for family-based genetic studies to better map inheritance patterns and phenotypic expression (1, 3). Diagnosis remains a rigorous process of exclusion, requiring the elimination of secondary causes such as Vitamin D deficiency, magnesium abnormalities, and renal insufficiency before proceeding to definitive GNAS gene testing (1). By utilizing whole-exome sequencing in a family-based cohort, this study aims to clarify the molecular mechanisms driving iPPSD, ultimately enhancing diagnostic accuracy and providing better understanding for the long-term clinical management of affected individuals and their families (1, 11).

## Clinical data of proband 1 and her family members

Proband 1 is a 37-year-old female farmer who presented with “recurrent perioral numbness and hand-foot numbness for 30 years, hand-foot cramps for 27 years, with worsening symptoms over the past 2 months.” Thirty years ago, she experienced recurrent perioral and hand-foot numbness, accompanied by perioral twitching and difficulty walking, as well as intellectual disability and smaller limb size compared to her peers. Twenty-seven years ago, she began experiencing intermittent hand-foot cramps, occasionally, these episodes were accompanied by foaming at the mouth and loss of consciousness, At a hospital visit, her blood tests revealed hypocalcemia and hyperphosphatemia. She received irregular treatment with vitamin D3 and calcium carbonate and continued to experience 1 to 2 episodes of hand-foot cramps per month. One year ago, she fell and injured her forehead after losing consciousness due to hand-foot cramps, she was admitted to our hospital, where laboratory tests showed elevated PTH, decreased calcium(Ca), and increased phosphorus (P) levels.

**Figure 1:**
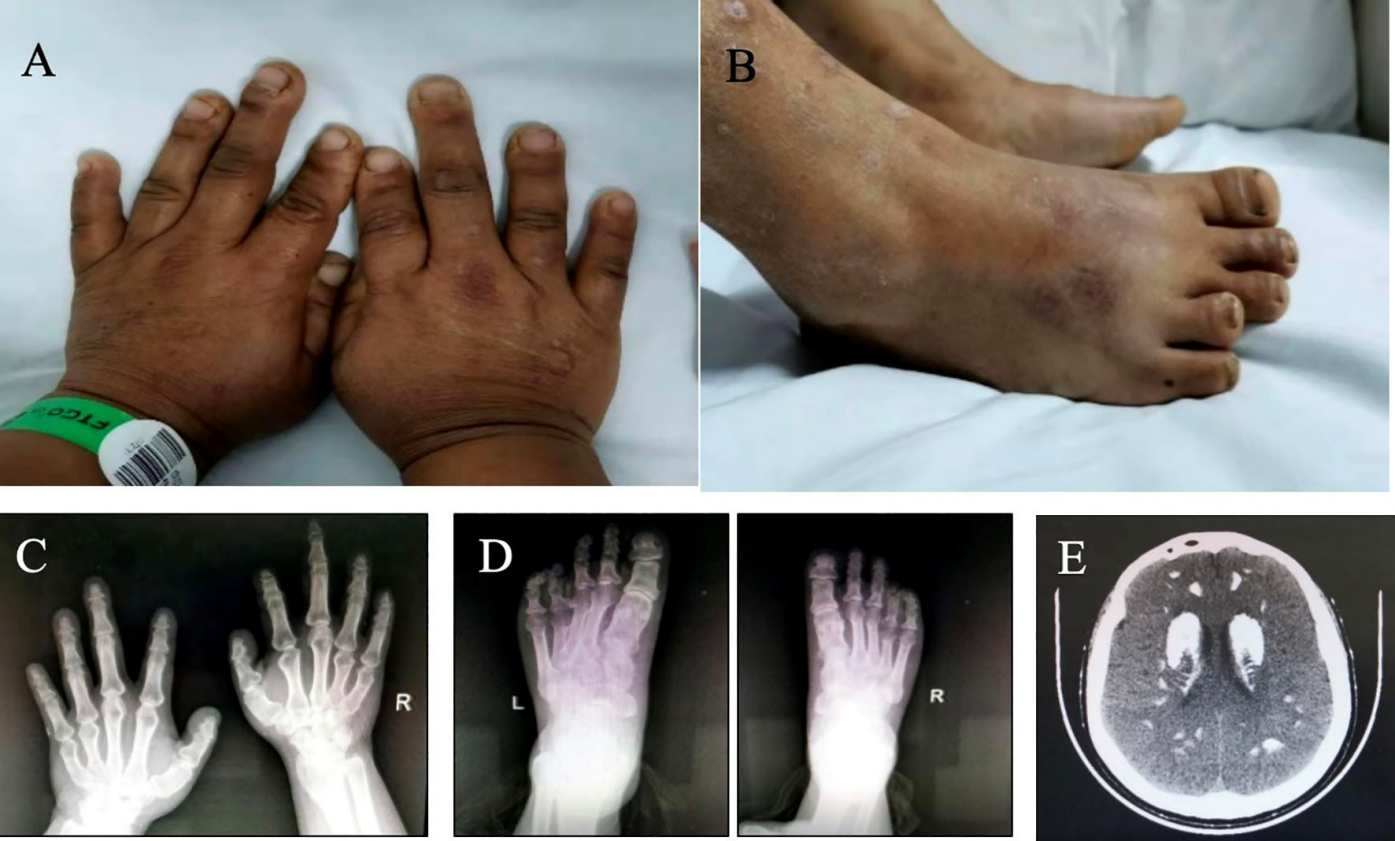
Appearance and imaging of proband 1. **(A)** hand appearance, **(B)** foot appearance, **(C)** X-ray of both hands, **(D)** X-ray of both feet, **(E)** CT of the head.

The patient is 150 cm tall, with a body mass index (BMI) of 26.2 kg/m (2). She displayed a slow response, a stocky stature, a round face, a flat nose, and a short neck. Her thyroid was not palpable. The fourth and fifth metacarpals of both hands were symmetrically shortened, as were the fourth and fifth phalanges of both feet (**Figures 1A–D**). The Trousseau sign was positive.

Blood tests revealed decreased Ca, increased P, and elevated PTH levels (**Table 1**). Free triiodothyronine (FT_3_) and free thyroxine (FT_4_) were within normal ranges, but TSH levels were elevated (**Table 1**). Gonadal hormones were consistent with follicular phase changes, and growth hormone levels were normal. A head CT scan revealed symmetrical calcification in the subcortical regions of both cerebral hemispheres, bilateral basal ganglia, thalamus, cerebellar hemispheres, and multiple intracerebral calcification foci (**Figure 1E**).

**Table 1:** Blood PTH, Ca, P and armour function levels of proband 1 and her family members.

Parameter	Proband 1	Mother	Sister	Son	Normal reference range
PTH (pg/ml)	193.5	41.4	35.77	542.6	16-65
Ca (mmol/L)	1.58	2.54	2.45	1.95	2.02-2.6
P (mmol/L)	1.63	1.04	1.2	2.95	0.87-1.45
TSH (μIU/ml)	5.443	0.905	2.838	9.655	0.38-4.34
FT_3_ (pmol/L)	4.70	4.86	5.04	7.62	2.77-6.31
FT_4_ (pmol/L)	13.34	13.7	14.6	13.2	10.45-24.38

PTH, parathyroid hormone; Ca, calcium; P, inorganic phosphorus; TSH, thyrotropin; FT_3_, free triiodothyronine; FT_4_, free thyroxine; the same below.

**Figure 2:**
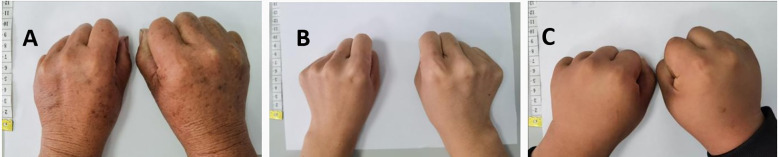
Hands of Proband 1 mother, sister and son. **(A)** Proband 1 mother, **(B)** Sister of proband 1, **(C)** Son of proband 1.

In Case 1, the proband’s parents are non-consanguineous. The father had passed away, and the mother, who is 150 cm tall, exhibits shortened fourth and fifth metacarpal bones in both hands (**Figure 2A**). However, her blood levels of PTH, Ca, P, and thyroid function were all normal (**Table 1**). A head CT revealed demyelination in the brain’s white matter and multiple calcification foci in the scalp. The proband’s sister has normal hand morphology (**Figure 2B**), and her blood PTH, Ca, P, and thyroid function (**Table 1**), as well as her head CT scan, were all normal. The proband’s son, a 12-year-old boy, weighed 3,200 g at birth and currently has a height of 148 cm. He has poor academic performance. Four years ago, he was found to have hypocalcemia, and like his mother, his fourth and fifth metacarpals were shortened (**Figure 2C**), as well as the fourth and fifth phalanges of both feet. His blood PTH levels were elevated, while his Ca and P levels were within normal range (**Table 1**). His TSH level was elevated (9.655 μIU/ml), although FT_3_ and FT_4_ were normal (**Table 1**), and both anti-thyroid peroxidase and anti-thyroglobulin antibodies were negative. A head CT scan revealed Ca deposits in the brain. Gonadal hormone and growth hormone levels were normal across the family members.

## Clinical data of proband 2 and her family members

Proband 2 is a 5-year, 9-month-old girl who was admitted to the hospital due to “hypocalcemia for 8 months and seizures for 1 month.” Eight months prior, she was found to have low blood Ca levels, and her parents started giving her Ca supplements. In the past month, the child experienced more than 10 seizures while awake. She was diagnosed at another hospital with hypocalcemia, hyperphosphatemia, and elevated serum PTH levels. After receiving Ca and alfacalcidol, her seizures did not recur.

**Figure 3:**
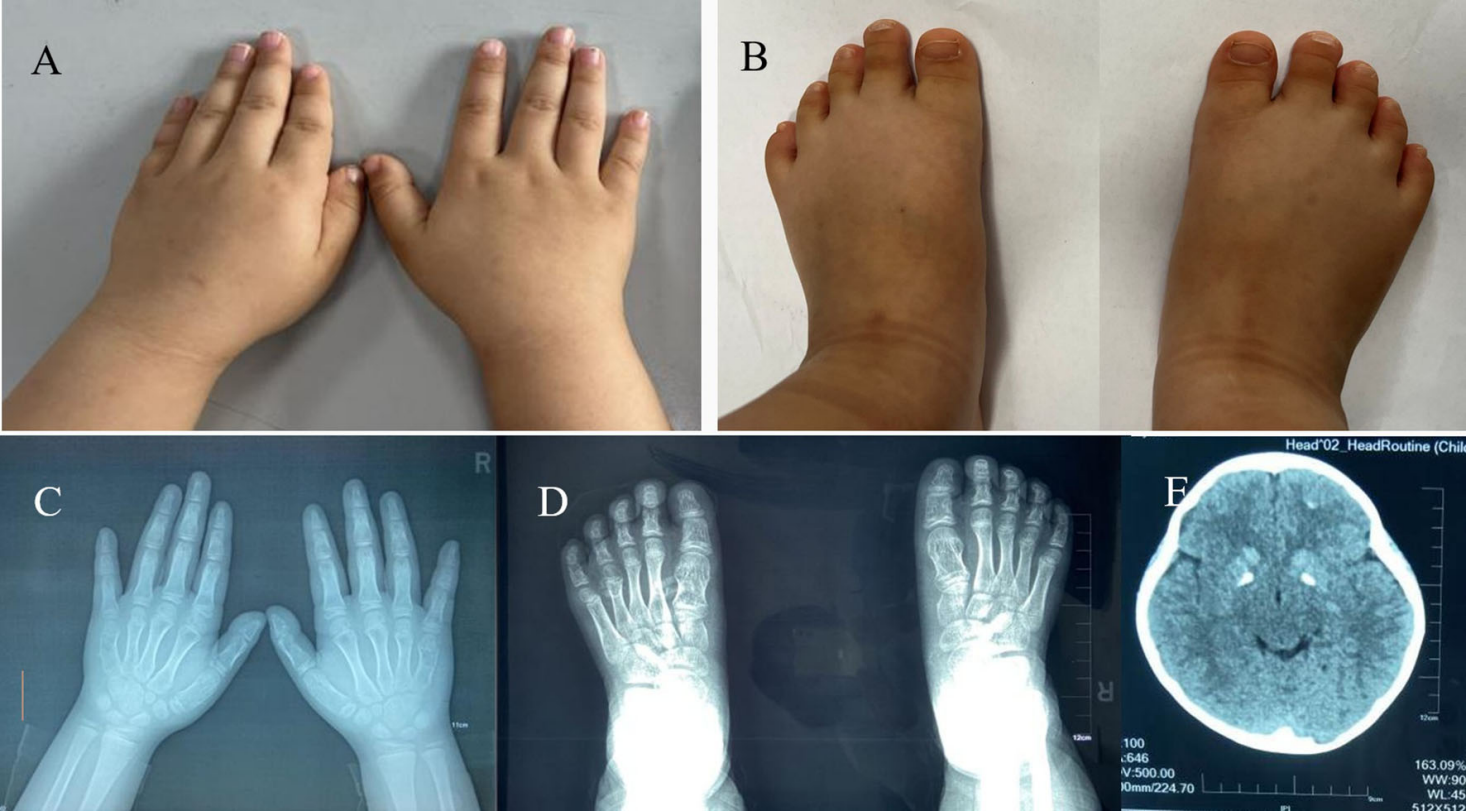
Appearance and imaging of proband 2. **(A)** Hand appearance, **(B)** foot appearance, **(C)** X-ray of both hands, **(D)** X-ray of both feet, **(E)** CT of the head.

The child’s height is 114 cm (around the 50th percentile for children of the same sex, age, and race), and her BMI is 18.1 kg/m^2^. She had slightly rough skin, a round face, and a flat nose. The Chvostek sign was negative, while the Trousseau sign was positive. Her fourth and fifth metacarpal bones in both hands were shortened, as were the fourth and fifth metatarsal bones in both feet (**Figures 3A–D**).

Blood tests revealed decreased Ca levels, elevated P, and high PTH levels (**Table 2**). Her TSH level was elevated (**Table 2**), and both anti-thyroid peroxidase and anti-thyroglobulin antibodies were negative. Sex hormone and growth hormone levels were within normal ranges. A head CT scan showed multiple calcifications in the bilateral basal ganglia and frontal cortex (**Figure 3E**).

**Table 2:** Blood PTH, Ca, P and armour function levels of proband 2 and her family members.

Parameter	Proband 2	Grandfather	Grandmother	Mother	Father	Normal reference range
PTH (pg/ml)	354	30.09	51.59	44.8	40.70	16-65
Ca (mmol/L)	0.89	2.43	2.33	2.37	2.35	2.02-2.6
P (mmol/L)	2.71	1.35	1.17	1.05	0.85	0.87-1.45
TSH (μIU/ml)	36.09	2.02	2.01	2.76	0.80	0.38-4.34
FT_3_ (pmol/L)	4.60	4.72	4.11	4.45	5.48	2.77-6.31
FT_4_ (pmol/L)	13.46	17.34	19.28	19.45	16.95	10.45-24.38

The proband’s father, mother, maternal grandfather, and maternal grandmother exhibit no clinical signs of hypocalcemia and no Albright’s hereditary AHO phenotype. Blood tests for PTH, Ca, and P levels were all within normal ranges (**Table 2**).

### GNAS genetic testing

Genomic DNA was extracted using the MagPure Buffy Coat DNA Midi KF Kit. The extracted DNA underwent enzyme digestion, screening, amplification, purification, and subsequent comparison with standard reference sequences. Sequencing results were viewed using Chromas software, and the gene sequence data were sourced from the NCBI database. Library construction was performed by Huada Biotechnology Co., LTD. Nucleotide sequence comparisons and amino acid sequence translations were conducted using DNAMAN software.

### Gene sequencing results of the probands and their family members and GNAS gene mutation analysis

**Figure 4:**
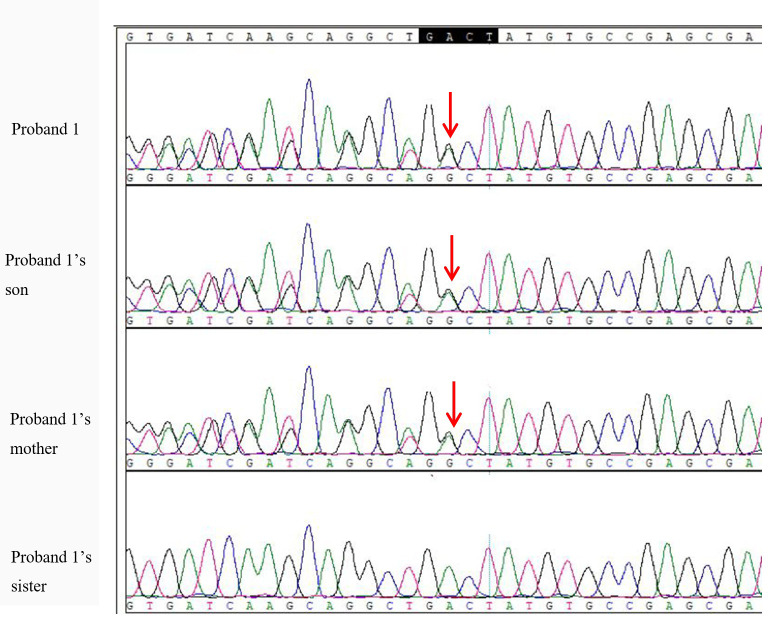
Peak nucleotide sequences of GNAS mutation sites in proband 1 and her family members. The arrow indicates nucleotide 568 of exon 7 at the starting mutation site, with double peaks visible on the Sanger.

The variant NM-001077488.2:c. 568571delGACT (p.Asp190Metfs*14) in the GNAS gene was detected in the peripheral blood of Proband 1, as well as in her mother and son. This mutation is classified as pathogenic for PHP. Proband 1, her mother, and her son all share the same heterozygous deletion of the nucleotides GACT at positions 568-571 in exon 7 of the GNAS gene, resulting in a frameshift mutation (**Figure 4**). According to the Human Genome Variation Society (HGVS) guidelines for gene mutation nomenclature, this mutation is denoted as GNAS: NM-001077488.2: c.568-571del GACT (p.Asp190Metfs*14).

**Figure 5:**
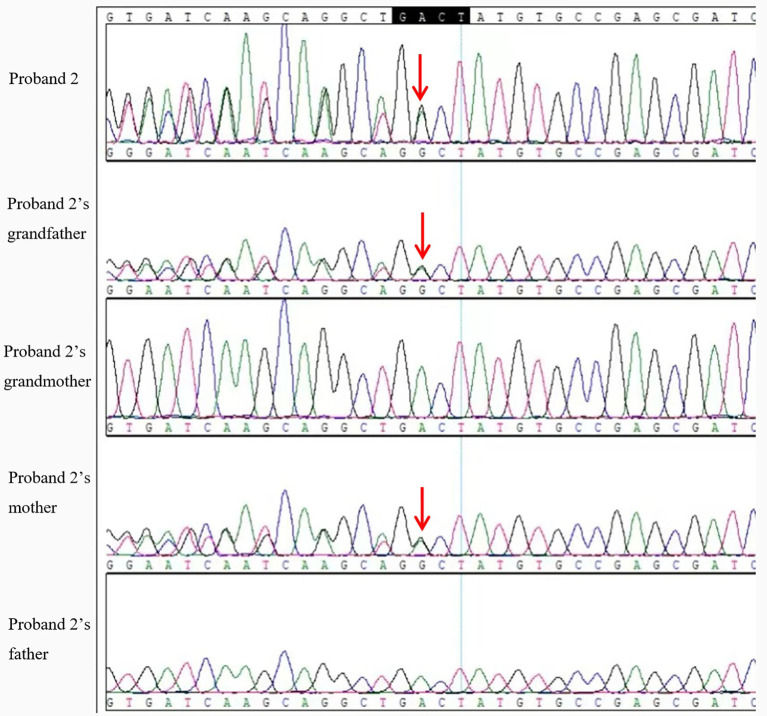
Peak nucleotide sequence of GNAS mutation site in proband 2 and her family members. The arrow indicates nucleotide 568 of exon 7 at the starting mutation site, with double peaks visible on the Sanger peak map.

The variant NM-001077488.2:c. 568571delGACT (p.Asp190Metfs*14) in the GNAS gene was detected in the peripheral blood of Proband 2, her maternal grandfather, and her mother. This mutation is classified as pathogenic for PHP. All three individuals share the same heterozygous deletion of the nucleotides GACT at positions 568-571 in exon 7, resulting in a frameshift mutation (**Figure 5**). According to the HGVS nomenclature rules, this mutation is denoted as GNAS: NM-001077488.2:c.568-571del GACT (p.Asp190Metfs*14).

### Sequence analysis of amino acid expression after mutation

**Figure 6:**
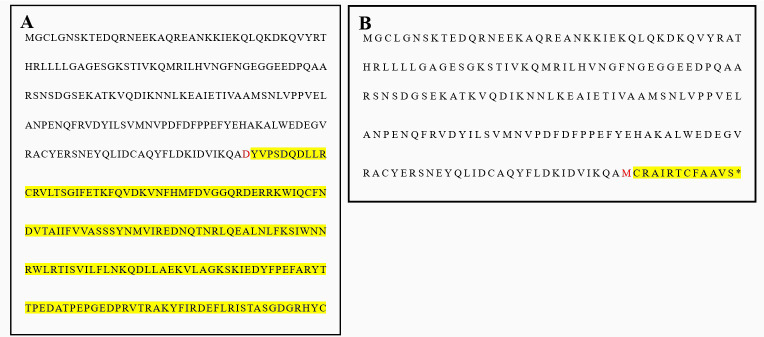
Wild and progenitor amino acid sequences of exon 7 of GNAS gene. **(A)** GNAS gene exon 7 wild amino acid sequence, amino acid 190 should be translated as aspartic acid [(D) for short]; **(B)** The amino acid sequence of proband 1 and 2, amino acid 190, which should have been translated as aspartic acid (D for short), was mutated and mistranslated as methionine (M for short), after which the translation of the amino acid sequence was erroneous and the translation was terminated prematurely.

Both Proband 1 and Proband 2 have the same GNAS gene mutation, NM-001077488.2:c. 568-571delGACT (p.Asp190Metfs*14), which alters the amino acid sequence downstream of the mutation site. Normally, amino acid 190 is translated as aspartic acid (abbreviated as D) (**Figure 6A**). However, due to the mutation, it is incorrectly translated as methionine (abbreviated as M) (**Figure 6B**). This frameshift mutation creates a new reading frame, causing premature termination of translation at codon 14 downstream of codon 190. As a result, the length of the original amino acid sequence is significantly shortened.

## Results of the family survey

**Figure 7:**
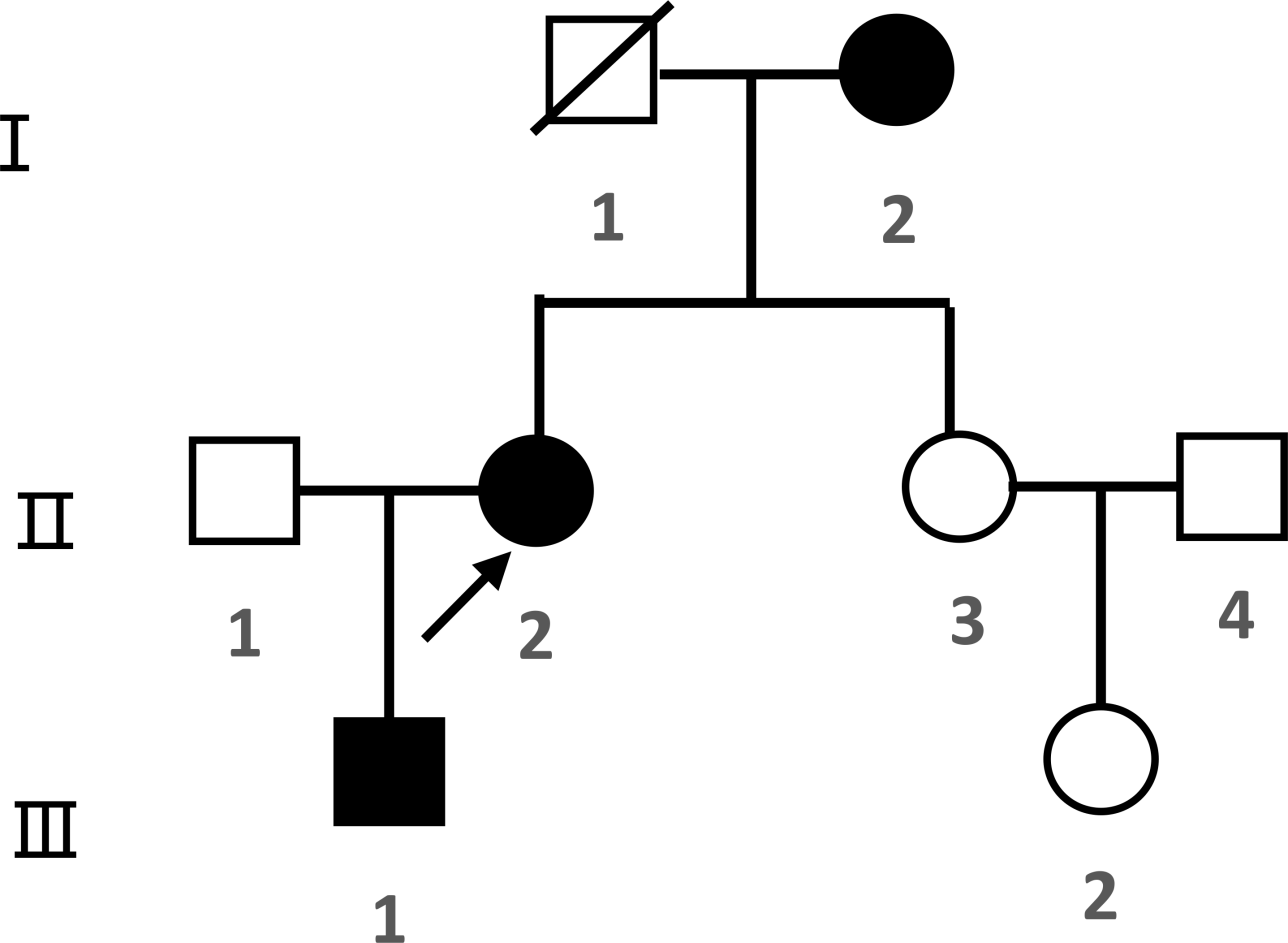
Family diagram of proband 1. I, II, III are generation numbers, 
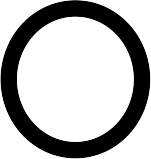
 normal female, 
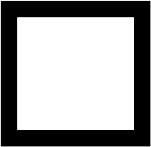
 normal male. 
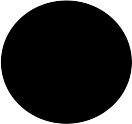
 female PHP patients, 
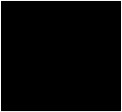
 male PHP patients, 
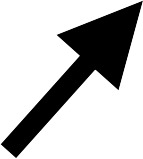
 progenitors, 
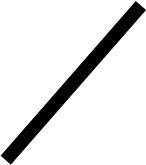
 has passed away, the same below.

Family diagram of proband 1 (**Figure 7**).

**Figure 8:**
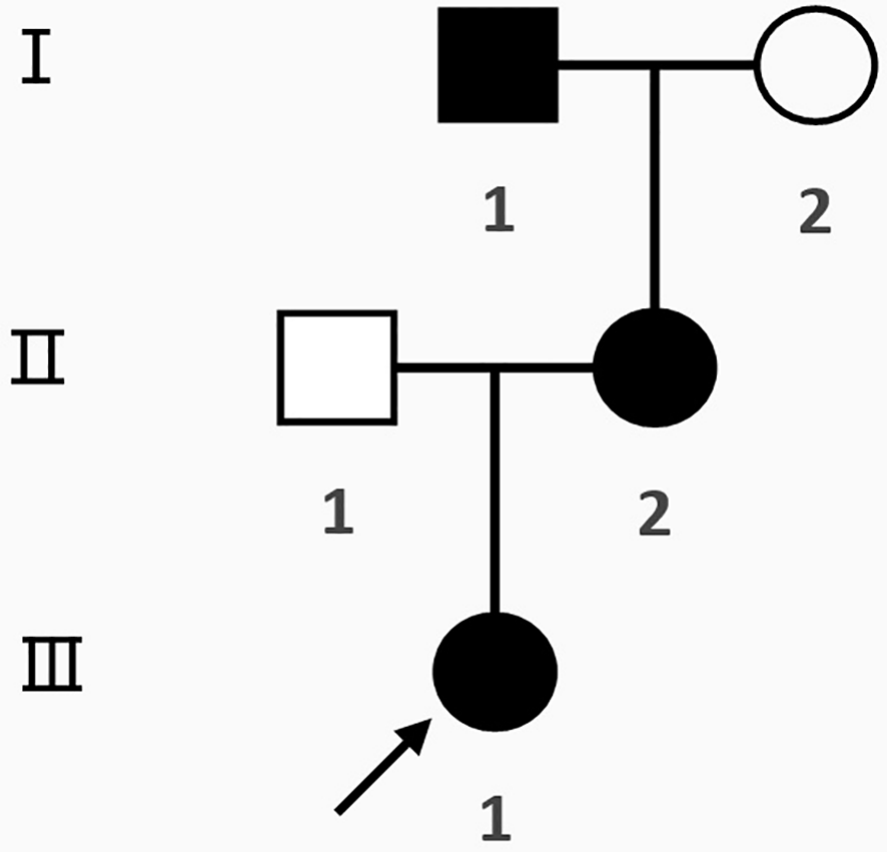
Family diagram of proband 2.

Family diagram of proband 2 (**Figure 8**).

### Diagnosis and treatment

**Figure 9:**
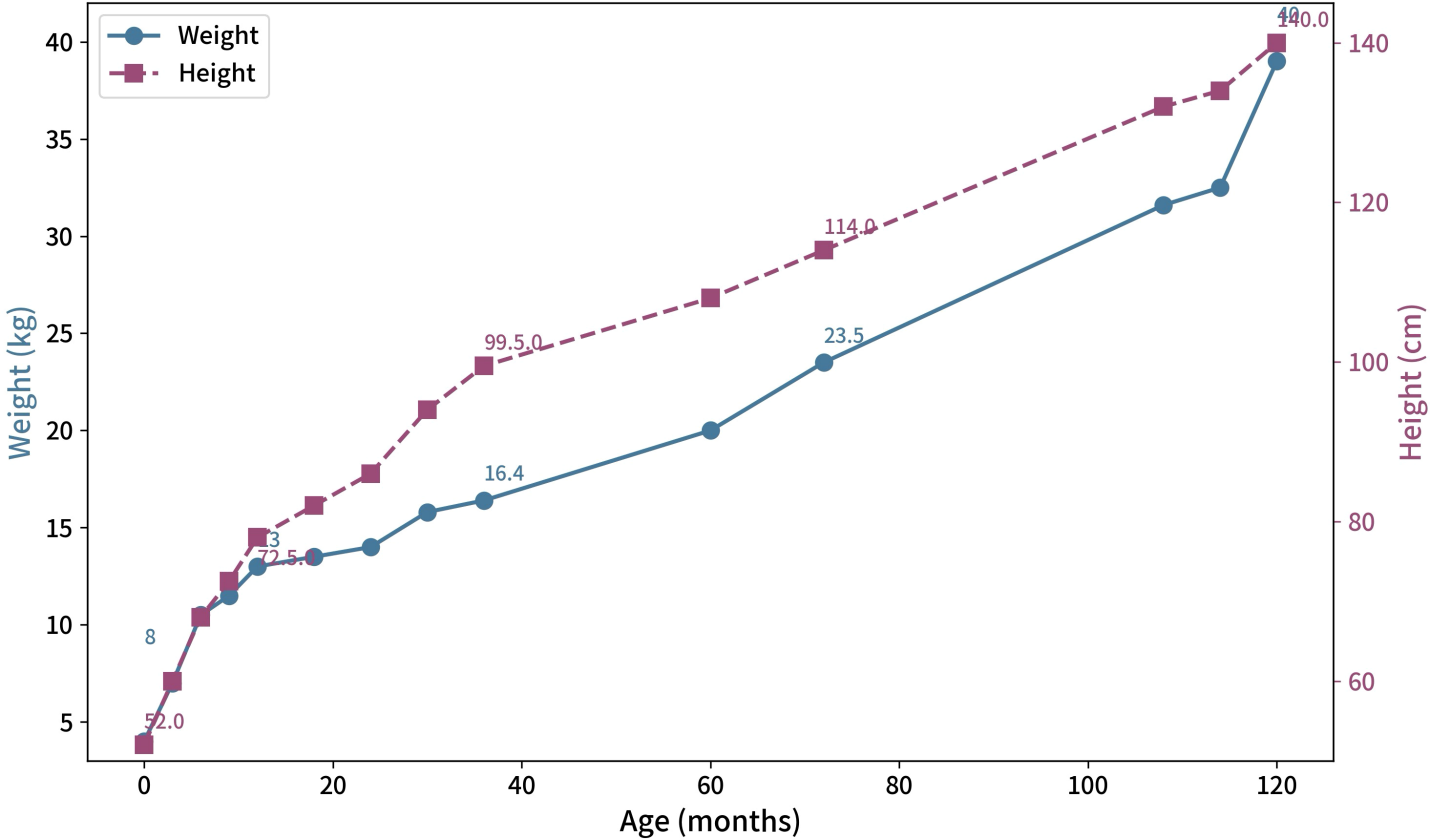
The growth curve of proband 2.

Proband 1 and her son, as well as Proband 2, were diagnosed with PHP type Ia, which belongs to iPPSD2. while Proband1’s mother, along with Proband 2’s maternal grandfather and mother, were diagnosed with PPHP, which also falls under the category of iPPSD2. In addition to lifestyle modifications, Proband 1, her son, and Proband 2 were treated with Ca and calcitriol (specific dosages are provided in **Table 3**). After more than six months of treatment, Proband 1 and Proband 2 experienced no further hand or foot spasms or seizures, and their blood levels of PTH, Ca, and P returned to normal. Proband 1’s son displayed no clinical symptoms of hypocalcemia, and his blood Ca level was normal, although his PTH and P levels remained elevated (**Table 3**). The growth curve of proband 2 is shown in **Figure 9**. At present, the height is between the 50th and 75th percentiles for girls of the same age, and the weight is at the 90th percentile.

**Table 3:** Evaluation of medication and blood efficacy of patients.

Subject	Therapeutic drug		PTH (pg/ml)	Ca (mmol/L)	P (mmol/L)
	Ca (mg/d)	Calcitriol (μg/d)			
Proband 1	600	0.5	41.4	2.54	1.04
Proband1’s son	600	0.5	340.7	2.15	2.3
Proband 2	180	0.5	51.25	2.46	1.45
normal reference range	——	——	16-65	2.02-2.6	0.87-1.45

## Discussion

In this study, both Proband 1 and Proband 2 sought medical attention due to the clinical manifestations of hypocalcemia. Proband 1, her mother, her son, and Proband 2 all exhibited AHO features. In addition to binding to PTH, PTH receptors also bind to PTH-related peptides, which are involved in endochondral bone development. Therefore, PHP patients may experience premature epiphyseal fusion in long bones, contributing to both the short finger (toe) deformities and the short stature observed in these individuals.

IPPSD2 initially reported by Albright in 1942, is inherited in an autosomal dominant manner and is the most common form of iPPSD. The pathogenesis involves a loss-of-function mutation in the maternal GNAS gene, with the most common mutation being a frameshift mutation. In addition to PTH resistance, patients with IPPSD2 often exhibit resistance to other hormones as well (12). The maternal GNAS gene mutation disrupts the PTH signaling pathway, resulting in reduced cAMP production, an essential second messenger in multiple hormone signaling pathways. Consequently, patients with iPPSD2 may develop resistance to several hormones, including TSH, LH, FSH, GHRH, catecholamines, and leptin (13, 14).

Currently, there is no curative treatment for iPPSD, and management primarily involves Ca and vitamin D supplementation. In addition to increasing dietary Ca intake, patients with PHP are advised to take Ca supplements, aily supplementation of 500-1000 mg of elemental Ca, and supplement with active forms of vitamin D, the recommended dose is 0.25-2μg per day of calcitriol (1, 25-dihydroxyvitamin D). Alternatively, 1α-hydroxyvitamin D may be given at a dose of 0.5-3.0 μg per day, or doxercalciferol at a dose of 0.2-1.0 mg per day. For patients with severe hyperphosphatemia, it is important to limit dietary P intake, particularly from sources such as dairy products and meats. Additionally, phosphate binders can be administered to help manage hyperphosphatemia. Throughout treatment, regular monitoring of blood PTH, Ca, P levels, and urinary Ca excretion is essential. Blood Ca levels should be maintained within or slightly below the normal range, vitamin D levels should remain in the normal range, and P levels should be within or slightly above normal. PTH should be controlled within or slightly above the normal range, as the distal renal tubules still retain some responsiveness to PTH, allowing for Ca reabsorption. Maintaining slightly elevated PTH levels can help reduce the risk of hypercalciuria.

In this case report, Proband 1 had been receiving irregular Ca and vitamin D supplementation prior to seeking medical attention, which led to recurrent symptoms. After being diagnosed with PHP and initiating regular treatment with Ca and calcitriol at our hospital, the patient experienced no further seizure episodes, and follow-up tests showed normal levels of PTH, Ca, and P. Proband 1’s son also received Ca and calcitriol treatment. While his blood Ca levels normalized, his PTH and P levels remained significantly elevated. He was advised to use phosphate binders, but his parents were reluctant to initiate the medication. Instead, they were instructed to control his diet, closely monitor his condition, and maintain a Ca-P product below 4.4 mmol^2^/L^2^. Proband 2 also received Ca and calcitriol treatment, and no further seizure episodes occurred. Follow-up testing showed that her PTH, Ca, and P levels were within the normal range. None of the three patients required high doses of Ca, and the calcitriol dose was limited to 0.5μg/day, which resulted in favorable treatment outcomes. These cases highlight the importance of regularly monitoring PTH, Ca, and P levels in iPPSD patients and gradually adjusting Ca and vitamin D supplementation to avoid excessive intake, which can lead to hypercalcemia or kidney stone formation. Additionally, Proband 1’s son, who is obese and has insulin resistance, was advised to manage his diet, increase physical activity, and remain vigilant for metabolic complications.

Patients with iPPSD2 often exhibit mildly elevated TSH levels at birth, without thyroid enlargement or the presence of thyroid-related antibodies (15). In the cases reported in this study, there were no clinical signs of hypothyroidism, Proband 1, her son, and Proband 2 all showed slightly elevated TSH levels, indicating potential TSH resistance. necessitating continued monitoring of thyroid function, thyroid-related antibodies, and ultrasound evaluations. If clinical hypothyroidism develops, thyroid hormone replacement therapy should be initiated. Long-term monitoring of thyroid function and thyroid-related antibodies is recommended. The other family members had normal thyroid function and showed no evidence of TSH resistance.

In addition to TSH resistance, most patients with iPPSD2 also experience GHRH resistance. In this study, due to limited testing conditions, GHRH levels were not measured, so it remains unclear whether the probands or their family members have GHRH resistance. We monitor the growth of Proband 2, her growth rate is greater than 6 cm per year, her height is between the 50th and 75th percentiles for girls of the same age, growth hormone therapy is not required temporarily. If growth retardation is observed, a GH stimulation test should be conducted to evaluate for GHD. If diagnosed GHD, rhGH replacement therapy should be considered (16). LH and FSH resistance can lead to gonadal dysfunction and incomplete sexual maturation, with female patients often experiencing amenorrhea or oligomenorrhea (15). In this study, Proband 1 had FSH and LH levels consistent with the follicular phase, while the other study subjects had normal FSH and LH levels, so hormone resistance of this type was not considered.

Since family members of a confirmed patient are likely to also have iPPSD, conducting a family investigation is essential. As iPPSD is predominantly an autosomal dominant genetic disorder, it is recommended that patients with PHP undergo GNAS gene testing as part of prenatal screening to ensure the health of future generations (17). In this study, the first diagnosed patient was identified at a relatively late stage, which impacted her social functioning and resulted in a poorer prognosis. In contrast, the younger age of Proband 1’s son and Proband 2 highlights the importance of early treatment to prevent complications and improve their quality of life. Without early detection, as patients with PHP age, they are at risk of developing skeletal abnormalities, intellectual disabilities, and endocrine-metabolic disorders. The insidious onset of PHP means that clinical symptoms often appear after laboratory abnormalities have already developed. Due to limited public awareness of PHP, patients and their families may not recognize the seriousness of the disease, leading to delayed treatment.

The two families in this study offer a concise teaching illustration of pseudohypoparathyroidism, distinguishing hypoparathyroidism (deficient PTH secretion) from pseudohypoparathyroidism (peripheral PTH resistance). GNAS mutations exhibit an autosomal dominant pattern with a parent-of-origin effect: the maternal allele is expressed in tissues critical to calcium-phosphate homeostasis, while the paternal allele is silenced. Consequently, maternal inheritance leads to iPPSD1a (PHP Ia), characterized by PTH resistance and AHO, whereas paternal inheritance results in iPPSD1n (PPHP), presenting with AHO without hormone resistance. These cases directly illustrate how the parental origin of the mutation dictates the clinical subtype—the central teaching point.

The investigation of these two iPPSD patients and their families reaffirmed that the NM-001077488.2: c.568-571delGACT (p.Asp190Metfs*14) mutation in the GNAS gene is a common hotspot mutation among iPPSD patients. PHP type Ia and PPHP, both belonging to iPPSD2, can manifest in different generations within the same family, and individuals with the same GNAS gene mutation may exhibit different clinical features, metabolic profiles, and hormone resistance. This variation underscores the influence of epigenetic, genetic, and environmental factors on phenotype expression. Given iPPSD’s subtle clinical presentation, routine blood PTH testing and GNAS gene mutation screening for patients with low Ca, high P, and AHO body features are crucial for early diagnosis and treatment. Early intervention helps prevent delayed treatment and improves patient prognosis.

Proband 1’s mother exhibited AHO features but had normal blood PTH, Ca, and P levels with no signs of hormone resistance. Genetic testing confirmed that she carried the same GNAS mutation as Proband 1, consistent with a diagnosis of. Similarly, Proband 2’s maternal grandfather and mother had no clinical signs of hypocalcemia, no AHO features, and normal blood levels of PTH, Ca, and P. They showed no evidence of hormone resistance. However, genetic testing revealed a GNAS gene mutation inherited from the paternal side, resulting in a diagnosis of iPPSD. iPPSD is inherited in an autosomal dominant manner, characterized by normal PTH, Ca, and P levels. Its pathogenesis involves an inactivating mutation of the paternal GNAS gene, leading to reduced Gsα activity. Patients may exhibit AHO features but typically do not present with intellectual disability, obesity, or other forms of hormone resistance.

This mutation is characterized as a “hotspot mutation” among patients with PHP; however, GNAS mutations are exceedingly rare in the general population, with the incidence of PHP/PPHP estimated at approximately 1–2 cases per million individuals. Familial aggregation studies indicate that this mutation follows an autosomal dominant pattern of inheritance and displays phenotypic heterogeneity, wherein the same mutation may manifest as either PHP Ia or PPHP. This variability is linked to parental imprinting, which involves differential expression based on maternal or paternal inheritance. Currently, the literature lacks comprehensive frequency data for this mutation across diverse global populations. The influence of maternal genetic factors in iPPSDs is primarily attributed to the imprinting phenomenon associated with the GNAS gene. In target organs such as the kidney and bone, the maternal allele of the GNAS gene is active, whereas the paternal allele remains inactive. Consequently, maternally inherited GNAS mutations can result in functional deficiencies of the Gsα protein in these tissues, leading to PTH resistance and features characteristic of AHO (18).

The influence of paternal genetic factors on iPPSDs is relatively minor, yet remains noteworthy. Notably, mutations in the PRKAR1A gene, which are typically inherited from the paternal lineage, contribute to the manifestation of iPPSD3 (19). The PRKAR1A gene, situated at 17q24.2, is not subject to genomic imprinting; thus, mutations inherited paternally can lead to functional impairments in the regulatory subunits of protein kinase A in progeny, thereby impacting the PTH/PTHrP signaling pathway (20). Additionally, paternal environmental factors may play a role in the pathogenesis of iPPSDs. For instance, paternal habits such as smoking and alcohol consumption may influence gene expression in offspring by inducing epigenetic modifications in sperm (21).

## Data Availability

The datasets presented in this study can be found in online repositories. The names of the repository/repositories and accession number(s) can be found in the article/supplementary material.
